# Evaluation of BACE1 Silencing in Cellular Models

**DOI:** 10.4061/2009/257403

**Published:** 2009-07-14

**Authors:** Malgorzata Sierant, Katarzyna Kubiak, Julia Kazmierczak-Baranska, Masaki Warashina, Tomoko Kuwabara, Barbara Nawrot

**Affiliations:** ^1^Department of Bioorganic Chemistry, Centre of Molecular and Macromolecular Studies, Polish Academy of Sciences, Sienkiewicza 112, 90-363 Lodz, Poland; ^2^Organ Development Research Laboratory, National Institute of Advanced Industrial Science and Technology (AIST), Central 4, 1-1-1 Higashi, Tsukuba Science City, 305-8562 Ibaraki, Japan

## Abstract

Beta-secretase (BACE1) is the major enzyme participating in generation of toxic amyloid-beta (A*β*) peptides, identified in amyloid plaques of Alzheimer's disease (AD) brains. Its downregulation results in decreasing secretion of A*β*. Thus, BACE1 silencing by RNAi represents possible strategy for antiamyloid therapy in the treatment of AD. In this study, a series of newly designed sequences of synthetic and vector-encoded siRNAs (pSilencer, pcPURhU6, and lentivirus) were tested against overexpressed and endogenous BACE1 in several cell lines and in adult neural progenitor cells, derived from rat hippocampus. SiRNAs active in human, mouse, and rat cell models were shown to diminish the level of BACE1. In HCN A94 cells, two BACE1-specific siRNAs did not alter the expression of genes of BACE2 and several selected genes involved in neurogenesis (Synapsin I, *β*III-Tubulin, Calbidin, NeuroD1, GluR2, CREB, MeCP2, PKR), however, remarkable lowering of SCG10 mRNA, coding protein of stathmin family, important in the development of nervous system, was observed.

## 1. Introduction

Alzheimer's disease (AD) is a progressive brain disease affecting the elderly population, causing problems with memory, thought, and behavior. Approximately 2–5% of AD cases are familial (FAD), caused by autosomal dominant mutations in amyloid precursor protein (APP) or the presenilin (PS1, PS2) genes [1–3], while the majority of sporadic AD cases are not associated with any known mutations. The hallmarks of Alzheimer disease include intraneuronal neurofibrillary tangles, consisting of the hyperphosphorylated microtubule-associated protein Tau and extracellular deposits of filaments of 42-residue amyloid *β* (A*β*) peptide [4, 5]. A*β* deposits become increasingly fibrilar and gradually acquire the classical features of amyloid plaques [6]. A*β* is the product of sequential cleavage of APP by *β*- and *γ*-secretases [7, 8]. Nonharmful APP cellular processing by *α*- and *γ*-secretases results in a short, highly soluble, nonamylogenic p3 peptide [9]. In alternative amyloidogenic processing, APP is hydrolyzed by *β*- and *γ*-secretases, and three possible peptides (A*β*40, A*β*42, A*β*43) can be generated [10]. Variability of the cleavage site of *γ*-secretase is associated with mutations in the PS1 and PS2 genes [11]. A*β* peptides are secreted from the presynaptic terminal into the extracellular matrix, where fibrillary A*β* deposits are formed outside of neurons. Some evidences suggest that the A*β* aggregates are the critical factor which triggers a complex pathological cascade leading to neurodegeneration [12]. All strategies to lower brain A*β*42 levels should be therapeutically beneficial in AD treatment. Given that BACE1 is the initiating enzyme in A*β* generation, it is considered a prime target for drug in AD for reducing cerebral A*β* levels [13–15].

RNA interference (RNAi) is an eukaryotic regulatory mechanism that uses double-stranded RNA (dsRNA) for induction of posttranscriptional gene silencing by the sequence-specific hydrolysis of homologous mRNA [16, 17]. RNAi has already been proposed for therapy of neurodegenerative diseases, including amyotrophic lateral sclerosis (ALS) [18], spinocerebellar ataxia (SCA) [19], Huntington's disease [20], and Alzheimer's disease [21–23]. Using lentiviral vectors expressing siRNAs targeting BACE1, Singer et al. reduced the cleavage of APP at the beta site, lowered amyloid burden, and achieved amelioration of dendritic and synaptic pathology in the hippocampus of tested animals [24]. Other BACE1 silencing studies, performed *in vitro* in primary neurons derived from APP-transgenic mice, resulted in reduced production of APP fragments, CTFs and A*β* [25].

The present studies concern optimization of RNAi for effective silencing of overexpressed and endogenous BACE1 protein in the human/mouse/rat cell cultures. For that purpose we used original sequences of siRNAs coding synthetic siRNA duplexes as well as shRNA-encoding plasmids and lentiviral vector. Moreover, we analyzed the effects of the use of BACE1-specific siRNAs and, in consequence, BACE1 silencing on the expression of selected genes involved, for example, in adult neurogenesis.

## 2. Materials and Methods

### 2.1. Preparation of siRNA Duplexes

The synthesis of RNA oligonucleotides was performed according to the routine phosphoramidite approach [26], using LCA CPG glass support and commercially available nucleoside phosphoramidites (Glen Research). Oligonucleotide synthesis was performed with an Applied Biosystems 394 instrument under the conditions recommended by the manufacturer. Assembly of siRNA duplexes was performed by mixing the equimolar amounts of complementary sense and antisense oligoribonucleotides in PBS buffer, heating at 96°C for 2 minutes, followed by slow (2 hours) cooling to room temperature. The duplex structure of resulted siRNAs was confirmed in a 4% agarose gel.

### 2.2. Construction of Plasmid Vectors

Inserts for cloning into the pcPURhU6 plasmid (iGene Therapeutics, Inc., Japan) were generated by annealing two complementary 62-mer oligonucleotides containing (i) a 21 nucleotide (nt) sense strand and 21 nt antisense strand separated by loop, (ii) a stretch of five thymidines as the Pol III promoter termination signal, and (iii) both sides of the insert flanked by BspMI restriction site [27]. Additionally, the use of the following loop sequences was explored: GTGTGCTGTCC, TTCAAGAGA [28], CTTCCTGTCA, and TAGTGAAGCCACAGATGTA. Cloning into the pSilencer 2.0-U6 plasmid (Ambion Inc., Applied Biosystems) was performed according to the manufacturer's protocol.

### 2.3. Cell Line and Culture Conditions

HeLa (human, cervical carcinoma) cells were cultured in RPMI 1640 medium (Gibco, BRL, Paisley) supplemented with 10% heat-inactivated fetal bovine serum (FBS) (Gibco, BRL, Paisley), 100 U/mL penicillin, and 100 *μ*g/mL streptomycin (Polfa) at 37°C and 5%  CO_2_. SH-SY5Y (human, Caucasian, bone marrow neuroblastoma) cells were cultured in 50% F12 Nutrient Mixture (HAM) medium (Gibco, BRL, Paisley)/50% MEM (Gibco, BRL, Paisley), supplemented with 15% FBS and antibiotics (100 mg/mL streptomycin and 100 U/mL penicillin). HEK293 cells (human, embryonic kidney) and M15 cells (mouse, mesonephric epithelium) were cultured in Dulbecco's MEM (Sigma-Aldrich Co., Saint Louis, MO), supplemented with 10% FBS and antibiotics at 37°C and 5%  CO_2_. Before transfection, the culture medium was replaced with fresh medium, free of antibiotics. Transfections were performed using Lipofectamine 2000 (Invitrogen) at a 2 : 1 ratio and appropriate siRNAs or plasmids encoding shRNA were added. For dual fluorescence assay, HeLa cells were cotransfected with plasmid DNA pDsRed2-N1, 15 ng/well (BD Biosciences) and p-BACE-GFP [29], 70 ng/well, provided by Dr. Weihong Song (The University of British Columbia, Vancouver, Canada), and with siRNA (0.1–5 nM final concentrations) or plasmids (pSilencer-shRNA/pcPURhU6-shRNA 30 ng/well) dissolved in OPTI-MEM medium (50 *μ*L/well, Gibco). The cells were incubated for 5-6 hours and then medium with transfection mixture was replaced with the fresh, culturing medium with antibiotics. After 48-hour incubation at 37°C in atmosphere of 5%  CO_2_, the cells were washed three times with phosphate saline buffer (PBS) and lysed overnight with mixture of NP-40 buffer (150 mM NaCl, 1% IGEPAL, 50 mM Tris-HCl (pH 7), 1 mM PMSF and PBS (ratio 1 : 3) at 37°C). Cell lysates were used for fluorescence determination by a dual fluorescence assay, as described previously [30]. Shortly, fluorescence values of enhanced green fluorescent protein (EGFP) and red fluorescent protein (RFP) were measured using a Synergy HT reader (BIO-TEK); data quantification was performed using KC4 software. Excitation and emission wavelengths were as follows: GFP *λ*_Ex_ = 485/20 nm and *λ*_Em_ = 528/20 nm; RFP *λ*_Ex_ = 530/25 nm and *λ*_Em_ = 590/30 nm. The siRNA activity was calculated as the ratio of GFP to RFP fluorescence values, averaged over eight repetitions. The relative level of fluorescence (GFP/RFP) in control cells (transfected with pBACE-GFP, pDsRed2-N1 and control s-0 siRNA) was taken as the reference (100%).

### 2.4. RT-PCR Analysis

HEK293, M15, SH-SY5Y cells were transfected with plasmids encoding shRNA expression cassettes using Lipofectamine 2000 (Invitrogen) according to the manufacturer's protocol. The cells were collected 36 hours after transfection, lysed with TriPure Isolation Reagent (Roche Applied Science), and total RNA was extracted and analyzed by RT-PCR. For a single reaction, 1 *μ*g of total RNA was used as a template. Primers of the following sequences: for BACE1 (h/m/r): forward (633–652) TGTGGAGATGGTGGACAACC, reverse (993-1012), ATCTCAGCATAGGCCAGCCC; for GAPDH (h): forward GAGTCAACGGATTTGGTCGT, reverse TTGATTTTGGAGGGATCTCG; for GAPDH (m/r): forward GTGTGAACGGATTTGGCCGT, GAPDH (m) reverse TTGATGTTAGTGGGGTCTCG, GAPDH (r) reverse TTGATGTTAGCGGGATCTCG were synthesized in house. Commercially available primers were used for RT-PCR amplification of genes listed in Table 2. The reverse transcription and PCR amplification reactions were performed using OneStep RT-PCR Kit (Qiagen). RT-PCR reactions were performed according to the manufacturer's protocol. Amplified samples were analyzed by agarose gel electrophoresis and quantified using ImageQuant 5.0 software. The level of GAPDH mRNA was used as a reference.

### 2.5. Western Blot Analysis

Cell lysates obtained by TriPure Isolation Reagent (Roche Applied Science) were used for preparation of total protein fraction, according to manufacturer's protocol. Protein samples (20 *μ*g for each lane) were separated by 10% SDS-PAGE and semi-dry electroblotted to Immobilon P PVDF membrane (Millipore Corp., Mass, USA). Rabbit polyclonal anti-BACE1 (Santa Cruz Biotechnology Inc.) in dilution 1 : 300 and rabbit polyclonal anti-*β*-actin (Abcam) in dilution 1 : 5000 were used as primary antibodies. Membrane was incubated with diluted antibodies overnight at 4°C. As secondary antibody, goat antirabbit IgG conjugated to alkaline phosphatase (Zymed, San Francisco) in dilution 1 : 5000 was used. Membrane was incubated one hour at room temperature. Bound antibodies were visualized by reaction of alkaline phosphatase with chemiluminescence substrate (Millipore Corp.) at the visualization system (G-BOX, Syngene).

### 2.6. Lentivirus Vector Production

The packaging system was described previously [31]. 293T cells were cultured in 10 cm dish in Iscove's Modified Dulbecco's Medium (Gibco/Invitrogen), supplemented with 1% L-glutamine, 10% heat-inactivated FBS (Sigma) and 1% antibiotics (Gibco/Invitrogen). The TUHC Pcsc-SP-PW-EGFP vector [32] (20 *μ*g), coding for the shRNA sequence, and helper vectors, pMDL (12 *μ*g), Rev (6 *μ*g), and VSVG (8 *μ*g), were cotransfected into 293T cells using a calcium-phosphate method [33, 34]. 48 and 72 hours after transfection, culture medium was collected, pooled together, filtered, and concentrated by ultracentrifugation. Lentivirus particles were suspended in PBS and stored at 4°C until use. Virus titers were determined by transduction of 293T cells and enhanced GFP expression visualization using fluorescence microscopy.

### 2.7. Transduction of HCN A94 Cells

HCN A94 cells (adult rat hippocampal neural stem cells) were cultured as described [35]. The day before transduction, cells were passaged into 6 well plates. Then, the cells were transduced with appropriate recombinant lentiviruses: Lv-si-5, Lv-si-6, and control Lv-EGFP. After infection, the HCN A94 cells were cultured in conditions specific for neuronal differentiation: in F12 media containing 1% N-2 Supplement (Gibco BRL, Paisley) with 1 *μ*M retinoic acid (RA) and 5 *μ*M forskolin (Sigma). Four days after infection, level of mRNA of the screened proteins were determined by RT-PCR.

## 3. Results

### 3.1. Selection of Active siRNA/shRNA Sequences in Dual Fluorescence Assay (DFA)

**Figure 1:**
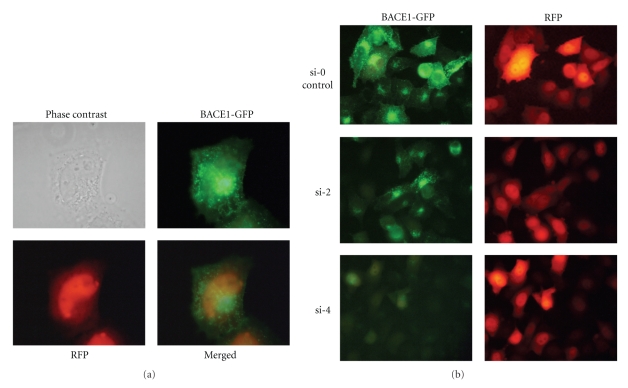
Expression of pBACE-GFP/pDsRed2-N1 plasmids in HeLa cells (a) and silencing effect of selected siRNAs in these cells (b).

Several target sites in human/mouse/rat (h/m/r) BACE1 genes were selected for siRNA duplexes using available tools [36–39]. The siRNA duplexes used had the typical structure of 19-base pairs (bp) fully complementary duplex with 2-nucleotide (nt) overhangs at each 3′ end, typically two thymidine units (Table 1). All RNA strands were synthesized in house and their structures and purity were uniformly confirmed by MALDI-TOF MS and PAGE electrophoresis, respectively, (data not shown). The shRNA encoding sequences were designed to express RNA transcripts with a hairpin structure of a 19-bp stem with a 9-nt loop: TTCAAGAGA (pSilencer 2.0-U6) [28], or a 21-bp stem with an 11-nt loop: GTGTGCTGTCC (pcPURhU6) [27]. To investigate the silencing activity of siRNA duplexes or shRNA-expressing vectors we optimized a dual fluorescence reporter system (DFA) [30, 40]. The system is based on measurement of the relative fluorescence intensity of enhanced green fluorescent protein (EGFP, expressed from fusion p-BACE-GFP [29] plasmid) and coral (*Discosoma spp.*) derived red fluorescent protein (RFP), expressed in HeLa cells from exogenously delivered plasmids. Localization of fluorescent proteins is shown in Figure 1(a), and interestingly BACE1-EGFP fusion protein localizes in the cytoplasmatic membranes, probably in the Golgi apparatus and/or ER compartments [41], while RFP protein is present in the nucleus and cytoplasm with no localization in the membrane. All duplexes used in the experiments mediated the human target gene silencing to varying extent (see Table 1), depending on the siRNA concentration and target site. Variability of siRNA potency is most likely the result of changed thermodynamic properties of the 5′- and 3′-ends of the duplexes as well as secondary structure of target mRNA [30]. Representative microscopic images of DFA control cells (transfected with pDsRed2-N1 and p-BACE-GFP plasmids) and cells additionally transfected with si-2 and si-4 duplexes are shown at Figure 1(b).

### 3.2. Activity of siRNA/shRNA Constructs Against Endogenously Expressed BACE1

**Figure 2:**
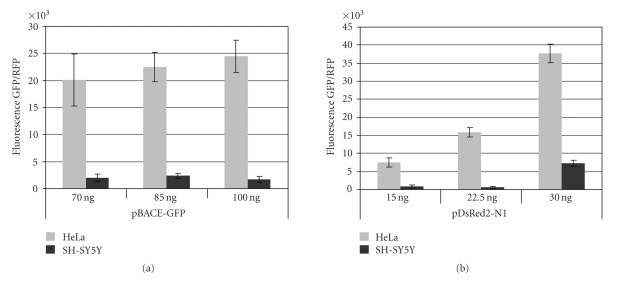
Comparison of transfection efficiency of pGFP-BACE or pDsRed2-N1 plasmids in HeLa and SH-SY5Y cells.

Silencing activities of siRNAs or shRNA-encoded plasmids directed toward endogenous BACE1 mRNA were screened in human (HEK293 and SH-SY5Y) and mouse (M15) cell lines by semiquantitative RT-PCR and by Western blot analysis. In SH-SY5Y cells, the most active was si-4 duplex which lowered BACE1 mRNA level up to 34% (100% value consists of BACE1 expression in control sample without silencing) (see Table 1). Low activity of siRNA in SH-SY5Y neuroblastoma cell line was probably caused by lower transfection efficiency of these cells as compared to HeLa cells, in which screened duplexes si-2–si-4 were much more active. Fluorescence of GFP or RFP expressed from pGFP-BACE or pDsRed2-N1 plasmids, respectively, was ca. 10–20 times higher in HeLa cells than in SH-SY5Y cells transfected in standard conditions (using lipofectamine at a 2 : 1 ratio) (see Figure 2). The pcPURhU6 si-5 vector (sequence corresponding to si-5a), specific for the human BACE1 gene, was the most potent inhibitor in HEK293 cells (32% of expression of the control sample). Additionally, pcPURhU6 si-5b, specific for mouse and rat BACE1 mRNA, also revealed high silencing efficiency in M15 mouse cells (30% of expression level of the control sample). The second plasmid construct, pcPURhU6 si-6 (sequence corresponding to si-6), specific for the h/m/r BACE1 gene, resulted in BACE1 expression of 51% in HEK293 cells and 71% in M15 cells.

### 3.3. Optimizing the Loop Length of shRNA Constructs

**Figure 3:**
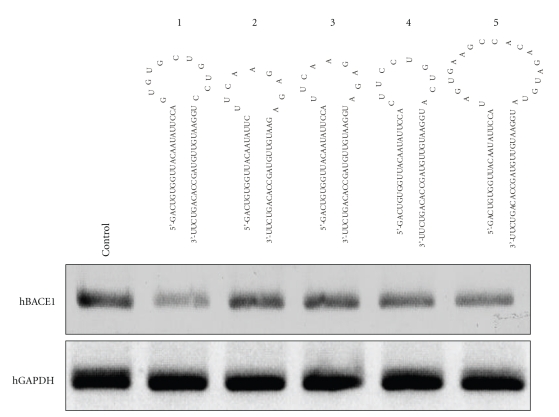
Optimizing of the loop sequence of si-6-based shRNA in HEK293 cells, as evaluated by RT-PCR: (1) pcPURhU6 si-6 (21 bp)-miR26, (2) si-6 (19 bp) with 9-nt UUCAAGAGA loop, (3) si-6 (21 bp) with 9-nt UUCAAGAGA loop, (4) si-6 (21 bp) with 10-nt CUUCCUGUCA (loop from miRNA23), and (5) si-6 (21 bp) with 19-nt UAGUGAAGCCACAGAUGUA (loop from miRNA30).

The loop structure has a significant impact on export of pre-miRNA or shRNA from nucleus [42] and their silencing efficiency was proven to be the most efficient with loops ranging from 9 to 19 nt [27, 28, 36]. We designed and cloned into the pcPURhU6 vector the hairpin-type RNAs with si-6 sequence (pcPURhU6 si-6) with the 19-21 base pair (bp) stems and with various loops: (1) pcPURhU6 si-6 (21 bp)-miR26, (2) si-6 (19 bp) with 9-nt UUCAAGAGA loop [28], (3) si-6 (21 bp) with 9-nt UUCAAGAGA loop, (4) si-6 (21 bp) with 10-nt CUUCCUGUCA (loop from miRNA23), and (5) si-6 (21 bp) with 19-nt UAGUGAAGCCACAGAUGUA (loop from miRNA30) (see Figure 3). All five plasmids were tested in HEK293 cells and the most active was the construct 1 causing 50% lowering of BACE1 mRNA. Constructs 4 and 5 with miRNA-origin loops miRNA23 and miRNA30, respectively, demonstrated moderate silencing activity, lowering BACE1 mRNA by 27% and 38%, respectively.

### 3.4. BACE1 Silencing in HCN A94 Progenitor Cells

**Figure 4:**
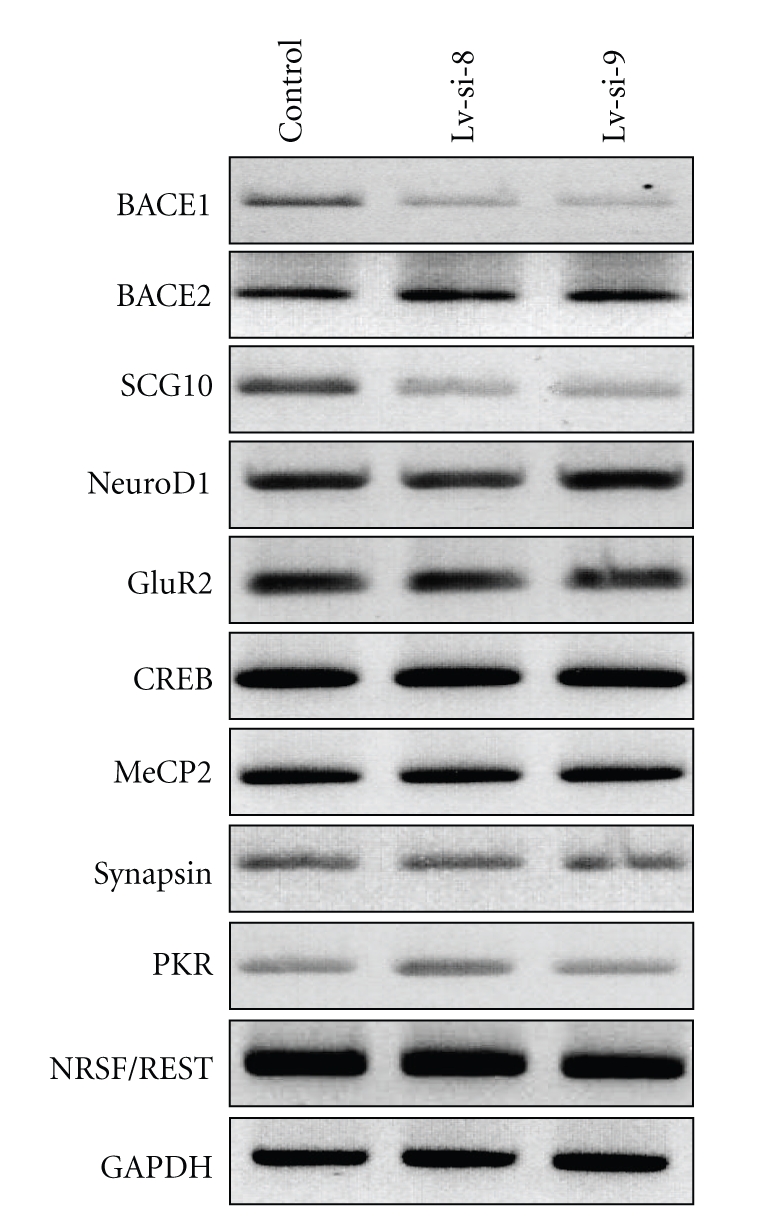
Influence of Lv-si-5b and Lv-si-6 and BACE1 silencing on expression of selected genes in HCN A94 cells, as determined by RT-PCR.

In order to validate the effects of shRNAs on BACE1 gene expression in rat cells model, the lentiviral vectors were constructed and used for the Lv-si-5 and Lv-si-6 lentiviruses production. The high titer lentiviruses Lv-si-5 and Lv-si-6 were used for transduction of adult hippocampal neural stem cells HCN A94. Both vectors caused significant reduction in BACE1 mRNA (>75%) in comparison to the level of mRNA expressed by control cells (Figure 4). In search for additional functions of BACE1 or products of BACE1 proteolytic activity, we analyzed mRNA levels of several genes involved in adult neurogenesis (listed in Table 2). Their list consists of a transcription factor Sox-2 (essential for maintaining progenitor cells in the undifferentiated stage), transcription factors Neurogenin 1–3 and NeuroD1, NRSF/REST (expressed during neuronal differentiation and responsible for activation of the expression of neuronal genes), Synapsin I, NaChII, GluR2, *β*III-Tubulin, Calbidin, BDNF, and SCG10. Additionally, we assessed expression of GFAP, indicative of astrocytes; MBP, specific for oligodendrocytes; and genes of CREB, MeCP2, APP, BACE2, PKR, and GAPDH. Selected data are shown in Figure 4. In most cases we did not find any influence of BACE1-specific siRNAs on the mRNA level of the above-mentioned genes. Interestingly, we found correlation between BACE1 and SCG10 gene expression, as BACE1 inhibition by Lv-si-5 and Lv-si-6 resulted in simultaneous downregulation of SCG10 (Figure 4, Table 2). Moreover, during neurogenesis in conditions favouring the differentiation of progenitor cells the level of BACE1 expression was increasing over the four days of the experiment (data not shown).

## 4. Discussion

Many Alzheimer's disease treatments are aimed at blocking the formation of pathogenic amyloid-*β* peptides. Both *β*- and *γ*-secretases, due to their crucial role in the secretion of A*β*, are supposed to be useful therapeutic targets. Successful inhibition of these enzymes may reduce the burden of amyloid-*β*-peptide in AD patients' brains, which may slow down the neurodegeneration. Inhibition of *γ*-secretase activity can evoke severe side effects, because this protease is involved in processing of other important substrates, including Notch receptor and cell surface proteins type I [43, 44]. Therefore, BACE1, being a major *β*-secretase participating in the toxic A*β* generation in the brain, is a primary therapeutic target. Multiple strategies have been used to inhibit BACE1 gene expression, including antisense oligonucleotides and catalytic nucleic acids [21, 25, 45–48]. In the present studies, we used the RNAi technology for selective inhibition of BACE1 gene expression. It was already demonstrated that silencing of BACE1 decreases the amount of secreted A*β* peptides [21, 24, 25, 47], therefore the main goal of these studies was to select siRNAs that are able to reduce specifically BACE1 expression in the designed experimental system. Several siRNA sequences directed toward human, mouse, and rat BACE1 mRNA were originally designed and their functionality against overexpressed and endogenous BACE1 was evaluated. The experiments were performed in HEK293 and SH-SY5Y human and M15 mouse cell lines as well as in HCN A94 rat cells, where a lentiviral vector expressing active shRNA was applied. As determined in the BACE-GFP/RFP dual fluorescence assay, the most active were si-3 and si-4 constructs, which at 100 pM concentration reduced the level of BACE-GFP mRNA by ca. 85%. These two siRNAs were the most active in the same system when introduced into pSilencer plasmid (5 and 11% of BACE1 expression, resp., in comparison to the control sample). Interestingly, two other siRNAs, si-5a and si-6, cloned into pcPURhU6 plasmid, silenced BACE1 in more than 80%, and may offer a good model system for further investigations with the use of plasmid-coded siRNA. Synthetic siRNAs, si-2, si-3, and si-4, transfected into human SH-SY5Y cells, reduced BACE1 mRNA and protein level to the lower extent than it was seen in DFA experiments performed in HeLa cells. Although SH-SY5Y neuroblastoma cells endogenously express high level of *β*-secretase and are often used in studies on neurodegeneration [49], their low transfection efficiency using conventional approach is the most limiting feature. 5-Fold lower transfection efficiency of pEGFP-C1 plasmid into SH-SY5Y cells in comparison to HeLa cells was observed with the use of conventional polycationic transfecting agents as well as with the more sophisticated gas plasma transfection methodology [50]. In our experiments, transfection of plasmid DNA, assessed by the extent of expression of pGFP-BACE or pDsRed2-N1 plasmids in SH-SY5Y cells, reached the level of only 10 –20% of that in HeLa cells (Figure 2).

siRNA si-5 was used in two forms—si-5a specific for human BACE1 mRNA and si-5b, varying with one nucleotide, specific for mouse and rat homologs. Both these siRNAs were rather selective toward cognate genes, as it is seen from experiments in HEK293 and M15 cells with siRNAs coded in pcPURhU6 plasmid (Table 1, the last column). Thus, fully complementary si-5a caused selective silencing of human BACE1 (32% of BACE1 expression) but being by one nucleotide mismatched (U→C) with mouse BACE1 mRNA, it was almost inactive toward this gene (92% of BACE1 expression). In contrary, the duplex si-5b, fully complementary to mouse BACE1 mRNA, was more efficient in mouse M15 cells than in HEK293 human cells. Such high specificity of siRNA with mutation at the position 16 of the antisense strand (counting from the 5′-end of this strand) was previously reported by Schwarz et al. for two alleles of the same gene of SOD1 [51].

To improve the knockdown efficiency of our constructs, we designed and cloned hairpin-type RNAs with the same stem sequence, but with various loops, derived from miRNAs: 23, 26b, or 30. All variants showed suppressive activity, but the most potent was the construct with the 21 bp stem and 11-nt loop from the microRNA 26b (50% expression of target BACE1). Moreover, the efficacy of constructs with the same loop sequence did not depend on the length of stem (19-bp or 21-bp) (see the activity of constructs 2 and 3, Figure 3). Finally, active siRNA sequences, si-5b and si-6, were cloned into lentiviral vector (TUHC pCSC-SP-PW-EGFP) and used to silence the target protein in rat adult neural progenitor cells HCN A94. Both lentivirus-coded siRNAs were very efficient in BACE1 silencing in HCN A94 cells, allowing to demonstrate that si-5b and si-6 duplexes, complementary to human, mouse, and rat mRNA target sites, can be simultaneously used as inhibitors of BACE1 gene expression in human, mouse, and rat cells, although with various efficiency in particular cells.

We asked a question, whether the activity of BACE1-specific siRNAs, and in consequence silencing of BACE1 protein, influence the profile of expression of other genes. Such nonspecific “off-target” effects are observed when target-specific siRNA molecules are used at high molar concentrations (>100 nM) [52, 53]. Subsequent analysis of mRNA levels of several genes involved in neurogenesis showed that screened siRNAs and BACE1 silencing did not affect the expression of most of selected genes (see Table 2). Unexpectedly, we observed downregulation of SCG10 expression. NCBI/BLAST analysis has shown no significant similarity between SCG10 and BACE1 sequences. Therefore, our observation suggests the presence of a regulatory pathway for SCG10 expression involving BACE1 or products of its proteolytic activity, although this hypothesis requires additional studies. SCG10 is a neuron-specific member of the stathmin family of microtubule regulatory proteins. Like stathmin, it can bind to soluble tubulin and depolymerize microtubules in neurons. Because this protein has restricted localization in neurons, expression of SCG10 gene is tightly regulated, mainly by NRSF/REST transcription factor [54]. Other regulators or modulators of SCG10 expression remain unknown. It would be interesting to explain the potential metabolic pathway between *β*-secretase and SCG10 and these studies are currently in progress. The SCG10 downregulation observed during RNAi-induced silencing of BACE1 may constitute important limitation of therapeutic application of this antiamyloid gene therapy, especially because increased expression of SCG10 is observed in the cortical and hippocampal regions of brain after the neuronal lesions [55].

In summary, we demonstrate high silencing activity of synthetic siRNAs and viral vectors-encoded shRNAs against overexpressed and endogenous BACE1. This process is fairly selective as expression of majority of screened genes involved in neurogenesis is not affected by BACE1-specific siRNAs nor by BACE1 level reduction.

## Figures and Tables

**Table 1:** siRNA sequences, target sites in human, mouse, and rat mRNA of BACE1 and silencing activity of used siRNA- and shRNA-coded plasmids (presented as % of BACE1 gene expression).

		Target gene (nt numbers)	% of BACE-GFP expression in DFA by siRNA (5/1/0.1 nM)*	% of BACE-GFP expression in DFA by shRNA in plasmid^∗#^	% of BACE1 expression in SH-SY5Y (human) cells by siRNA (RT-PCR/western blot)^∗&^	% of BACE1 expression in HEK293 (human)/M15 mice cells by shRNA in pcPURhU6^@^ (RT-PCR)
	Sequence:					
No	5′-sense strand-3′					
	3′-antisense strand-5′					
	
si-0	5′-AAUCAGAUUGAACCUUCAUTT-3′	non-silencing control siRNA	100	100	86	100
	3′-TTUUAGUCUAACUUGGAAGUA-5′					

si-1	5′-UACAGGCAGCAGUAACUUUTT-3′	**h** (733–753)m (-)r (-)	27/31/58	40 (pSilencer)	nd	nd
	3′-TTAUGUCCGUCGUCAUUGAAA-5′					

si-2	5′-AGACGCUCAACAUCCUGGUTT-3′	**h** (710–730) m (-)r (-)	6/22/76	nd	61/60	nd
	3′-TTUCUGCGAGUUGUAGGACCA-5′					

si-3	5′-GAAUCAGACAAGUUCUUCAUC-3′	**h** (946–966)m (-)r (-)	0.0/1.1/16	5 (pSilencer)	55/61	nd
	3′-GACUUAGUCUGUUCAAGAAGU-5′					

si-4	5′-AAUCAGACAAGUUCUUCAUTT-3′	**h** (947-967)m (-)r (-)	0.0/1.7/15	11 (pSilencer)	34/61	nd
	3′- TTUUAGUCUGUUCAAGAAGUA-5′					

si-5a	5′-AUCAGACAAGUUCUUCAUCAA-3′	**h** (948–968) m (-)r (-)	28/35/60	12 (pcPURhU6)	nd	32/92
	3′-GAUAGUCUGUUCAAGAAGUAG-5′					

si-5b	5′-AUCGGACAAGUUCUUCAUCAA-3′	h (-)**m** (920–940)**r** (920–940)	nd	nd	nd	52/30
	3′-GAUAGCCUGUUCAAGAAGUAG-5′					

si-6	5′-GACUGUGGCUACAACAUUCCA-3′	**h** (1777–1797)**m** (1751–1771)**r** (1751–1771)	1.3/8/45	20 (pcPURhU6)	nd	51/71
	3′-UUCUGACACCGAUGUUGUAAG-5′					

*: GFP/RFP relative fluorescence of the control, HeLa cells transfected with control nonsilencing siRNA (si-0) or pSilencer-Neg plasmid/Lipofectamine 2000 were taken as a reference nonsilenced value of 100%;

^#^: plasmid DNA 30 ng/well (96-well plate);

^&^: 100 nM siRNA;

^@^: plasmid DNA 2 *μ*g/well (6-well plate);

Nd: not determined.

**Table 2:** List of genes selected for expression level evaluation in HCN A94 cells transfected with BACE1-specific si-5b and si-6, coded in lentivirus vector.

No	Protein	Name/Function	% of silencing with Lv-si-8	% of silencing with Lv-si-9
1	BACE1	Beta-site APP cleaving enzyme 1	>70	>75
2	BACE2	Beta-site APP cleaving enzyme 2	—	—
3	SCG10	Superior cervical ganglion-10	70	70
4	NeuroD1	Neurogenic differentiation 1	—	—
5	GluR2	Glutamate receptor 2	—	—
6	CREB	cAMP responsive element binding protein 1	—	—
7	MeCP2	Methyl CpG binding protein 2	—	—
8	Synapsin 1	Protein involved in the regulation of neurotransmitter release at synapses	—	—
9	PKR	Double stranded RNA-dependent protein kinase	—	—
10	GAPDH	Glyceraldehyde-3-phosphate dehydrogenase	—	—
11	Sox-2	SRY (sex determining region Y)-box 2	—	—
12	NRSF/REST	Neural restrictive silencing factor	—	—
13	NaChII	Sodium channel, voltage-gated, type II	—	—
14	*β*III-Tubulin	Neuro-specific tubulin, microtubules component	—	—
15	Calbidin	Vitamin D-dependent calcium-binding protein	—	—
16	BDNF	Brain-derived neurotrophic factor	—	—
17	GFAP	Glial fibrillary acidic protein, protein specific for astrocytes in CNS	—	—
18	APP	Amyloid Precursor Protein	—	—
19	Neurogenin 1–3	Neurogenins, family of the transcription factors involved in the neuronal differentiation	—	—
